# Evolution of an Enzyme from a Noncatalytic Nucleic Acid Sequence

**DOI:** 10.1038/srep11405

**Published:** 2015-06-19

**Authors:** Rachel Gysbers, Kha Tram, Jimmy Gu, Yingfu Li

**Affiliations:** 1 Department of Biochemistry and Biomedical Sciences, McMaster University, 1280 Main St. W., Hamilton, ON L8S 4K1, Canada; 2 Department of Chemistry and Chemical Biology, McMaster University, 1280 Main St. W., Hamilton, ON L8S 4K1, Canada; 3 Origins Institute, McMaster University, 1280 Main St. W., Hamilton, ON L8S 4K1, Canada

## Abstract

The mechanism by which enzymes arose from both abiotic and biological worlds remains an unsolved natural mystery. We postulate that an enzyme can emerge from any sequence of any functional polymer under permissive evolutionary conditions. To support this premise, we have arbitrarily chosen a 50-nucleotide DNA fragment encoding for the *Bos taurus* (cattle) albumin mRNA and subjected it to test-tube evolution to derive a catalytic DNA (DNAzyme) with RNA-cleavage activity. After only a few weeks, a DNAzyme with significant catalytic activity has surfaced. Sequence comparison reveals that seven nucleotides are responsible for the conversion of the noncatalytic sequence into the enzyme. Deep sequencing analysis of DNA pools along the evolution trajectory has identified individual mutations as the progressive drivers of the molecular evolution. Our findings demonstrate that an enzyme can indeed arise from a sequence of a functional polymer via permissive molecular evolution, a mechanism that may have been exploited by nature for the creation of the enormous repertoire of enzymes in the biological world today.

Life would not have evolved and flourished on Earth without the arrival of enzymes, but the mechanism by which enzymes arose from the prebiotic world is an unsolved natural mystery. Life is purported to have surfaced beginning with the self-replication of ribonucleic acids in a hypothetical RNA world^1^,^2^,^3^,^4^,^5^. RNA is capable of storing information, and since the discovery of many natural^6^,^7^,^8^,^9^,^10^ and artificial^11^,^12^,^13^,^14^,^15^ RNA catalysts (ribozymes), the RNA world hypothesis has been found increasingly plausible. Even considering the RNA world hypothesis, the question remains: how did a ribozyme emerge in the RNA world? We postulate that a ribozyme can emerge from a noncatalytic sequence of RNA under permissive evolutionary conditions. To demonstrate this idea, we conducted a test-tube evolution experiment^16^,^17^,^18^ to convert a randomly chosen, noncatalytic sequence of single-stranded DNA, a proxy for RNA, into a catalytic DNA (DNAzyme) with RNase-like activity. Previous ribozymes and DNAzymes have been selected from libraries of completely random sequences^11^,^13^,^14^,^15^,^19^,^20^, or randomized versions of a sequence with a dissimilar function^12^,^18^. In contrast, our study aimed to establish a catalyst from a distinct, distant and non-catalytic corner of sequence space. After just weeks, a DNA pool with significant catalytic activity was established. High-throughput sequencing analysis has identified mutations that have enabled the noncatalytic to catalytic sequence conversion. Our findings demonstrate for the first time that an enzyme can arise from a defined sequence of a functional polymer via molecular evolution, a mechanism that may have been exploited by nature to initiate the evolution of enzymes in the RNA world and beyond.

Our approach is schematically demonstrated in Fig. 1a, designed to evolve a noncatalytic DNA strand into a DNAzyme capable of cleaving a single ribonucleotide embedded in a DNA sequence^19^. The starting sequence for the experiment was arbitrarily chosen to be the first 50 nucleotides (green nucleotides, Fig. 1b) of the *Bos taurus* (cattle) albumin gene (NCBI Reference Sequence: NM_180992.2; GI: 31340937). This candidate sequence was flanked on each side by an arbitrarily chosen 20-nucleotide sequence (grey nucleotides, Fig. 1b), which were intended as the primer-binding site for polymerase chain reaction (PCR) used to amplify the cleavage product and introduce a low level of point mutations into the evolving sequence. The 90-nucleotide DNA molecule is denoted BTA1. The substrate sequence, S1, contains 28 nucleotides (blue nucleotides, Fig. 1b) with a single ribonucleotide, guanosine ribonucleotide (purple G), located at the 14^th^ position. Both S1 and BTA1 were produced synthetically using automated DNA synthesis.

The steps involved in the *in vitro* evolution experiment are depicted in Figure S1 (Supporting Information). First, phosphorylated BTA1 (denoted generation 0 or G0) is ligated to S1, and the ligated S1-BTA1 is purified by denaturing polyacrylamide gel electrophoresis (dPAGE) before being subjected to metal-ion-promoted RNA-cleavage reaction for 4 hours. The cleaved BTA1 is purified by dPAGE, amplified by two consecutive PCR amplifications where the second PCR uses a modified primer that contains a hexaethyleneglycol spacer and A20 tail at the 5′ end. The spacer prevents the poly-A tail from being amplified, making the non-DNAzyme-coding strand 20 nucleotides longer than the coding strand. The design facilitates easy separation of the coding strand via dPAGE. The regenerated BTA1 molecules, some of which now contain low levels of mutations acquired during the PCR steps, are ligated again to S1 to initiate the second round of selection and amplification. This procedure is repeated as many cycles as needed until the BTA1 pool acquires the detectable catalytic activity.

No significant cleavage activity was observed for G0 through G20. However, a weak but noticeable cleavage activity was observed with G21, which continued to intensify for the next 5 cycles. By G25, a strong cleavage activity was established. We then amplified four DNA pools, G16, G19, G22 and G25 and subjected them to cleavage assay (Fig. 1c). While both G16 and G19 showed no detectable activity even after 72 hours of incubation, both G22 and G25 exhibited robust cleavage activities in a time-dependent manner. The cleavage activity was detectable in G25 even after 1 hour of incubation; by 72 hours, a significant fraction of G25 was cleaved. We compared the self-cleaving activities of S1, G0 and G25 using an extended time course (Fig. 1d). While the first-order cleavage rate constant of S1 and G0 was 5.5 × 10^−7^/min^21^, G25 had a rate constant of 4.3 × 10^−4^/min, corresponding to nearly 1,000-fold rate enhancement. These analyses indicate that we have successfully converted BTA1 into an RNA-cleaving DNAzyme.

The G25 pool was subjected to deep sequencing and the top 10 sequence classes, denoted G2501-G2510, are listed in Fig. 2a. As shown in the pie chart in Fig. 2b, the top class, G2501, represents 54.83% of the G25 pool; the second most-abundant variant, G2502, comprises 8.17% of the pool; the next 8 sequence classes collectively account for 12.77% (Fig. 2b). We compared the cleavage activities of the top 10 sequences and found that all of them exhibited similar cleavage activity, with the top class holding a slight edge over the other sequences (Fig. 2c).

Each of the top 10 classes acquired 6–8 mutations (including deletions) within the 50-nucleotide sequence element (Fig. 2a). Interestingly, most of the mutations appear to be linked to the selection pressure, rather than random drift, as they occur in all or most of the sequence classes. Most strikingly, the following 4 mutations are observed in all top 10 sequences: A13G, ΔC17, ΔC18 and T50C, suggesting that they play either essential or highly important roles in the noncatalytic to catalytic conversion. In addition, T6C, T39A and G40A are observed with most of the top 10 sequences, implying they act as favourable mutations during the catalyst evolution.

Upon inspection of the sequences of the top 10 classes, we propose a putative secondary structure for G2501 (Fig. 2d). The structure contains 5 short duplex elements: P1, P2a, P2b, P3 and P4. According to this structural model, four of the seven common mutations, A13G, T50C, T39A and G40A result in more stable P1, P2a and P4 duplex motifs. The general structure is supported by previously discovered RNA-cleaving DNAzymes, which consist of a catalytic motif flanked by two duplex-forming arms that bind to the substrate^19^,^20^. We further speculate that the catalytic core is made of P1, P2a and P3, along with the single-strand region located between P1 and P3, named SS1/3. If this is correct, the deletion of C17 and C18 might simply be an outcome of properly positioning the catalytic core for either the cleavage site recognition or chemical catalysis or both. Another well-known RNA-cleaving DNAzyme, 8–17, also contains a stem-loop with three base pairs followed by an unpaired region of 4–5 nt in its catalytic core^20^, similar to our proposed structure, which contains four base pairs followed by an unpaired region of seven nt.

We also examined the catalytic activity of several shortened G2501 variants each with 5-nucleotide deletion within the original 50-nucleotide sequence element and the data is provided in Figure S3. Deletions occurred within P2b and P4 did not affect the catalytic activity. However, the deletions made at P1, P2a, P3 and SS1/3 dramatically reduced the catalytic performance of the construct. Based on these observations, we derived a simplified secondary structure of G2501, which is shown as the first structural model in Figure S4. It is a trans-acting construct with perfectly matched P1 and P2 and the removal of all catalytically disposable nucleotides. To verify duplex elements within the putative secondary structure of G2501, we designed two more structural variants. In the first variant, the nucleobases within both P1 and P2 were significantly altered in an attempt to change the sequence identity but maintain the duplex structure. The second variant was designed on the same principle but targeted P3 for base-pair co-variations. The data provided in Figure S4 fully supports the proposed structural model.

We then investigated a potential evolutionary pathway between G0 and G2501. For this purpose, we conducted deep sequencing analysis of the generation 1, 2, 4, 6, 8, 10, 12, 14, 16, 17–25 pools. As there are a vast number of potential pathways between G0 (renamed MD0) and G2501 (renamed MD7), we have defined the sequence space used to propose a pathway as being comprised of all sequences possessing mutations required to mutate MD0 to MD7 with the minimum number of mutational events. We also assume that in a given round of selection, only a single mutation occurs, as single mutational events are more likely to occur than multiple simultaneous mutations. Figure 3 lists all possible sequences with 1–6 mutations along the proposed mutation pathway. For example, there are 6 single-mutation possibilities: T6C, A13G, G17- (or G18- as they are equal), T39A, G40A and T50C (second column, Fig. 3).

To determine a mutation pathway, we began by identifying mutants showing a strong signal throughout selection through calculating the proportion of each sequence in the sequence space across all sequenced rounds where a minimum of 3 copies are detected. The 4-mutation sequence A13G/C17-/C18-/T50C (named MD4) is detected in high proportion and persistently relative to other 24 4-mutation sequences. Using MD4 as an anchor we expand the mutation chain working in both directions (Fig. 3). Among 16 possible 5-mutation sequences, only 3 fall within the MD4 mutation chain, and of these, A13G/C17-/C18-/G40A/T50C (MD5) fits the best as it becomes persistent shortly after MD4 appears. Given MD5, only two 6-mutation sequences are possible. Mutant T6C/A13G/C17-/C18-/G40A/T50C (MD6) is persistent after the appearance of MD5, therefore we believe the 6^th^ mutation acquired to be T6C. This puts T39A as the final mutation.

Working backwards from MD4, A13G/C17-/C18- (MD3) shows the greatest persistence amongst the 3 possible mutants, suggesting T50C is the 4^th^ mutation. There are two 2-mutation mutants (C17-/C18- and A13G/C17-) that can give rise to MD3; we give C17-/C18- a favourable consideration as a deep sequencing analysis of the mutation distribution of the DNA polymerase showed a higher than expected proportion of sequential double deletions than would be expected for independent single deletion events (data not shown), therefore we believe it is likely that the C17-/C18- (MD2) double deletion occurred very early in the evolution. Based on the above analysis, we proposed the following mutation chain: C17- → C18- → A13G → T50C → G40A → T6C → T39A (Fig. 3).

Finally we analyzed time-dependent cleavage activity of each mutant sequence – MD0-MD7 – along the proposed evolutionary pathway (Fig. 4a). The activity profile provided in Fig. 4b indicates that two mutations along this proposed pathway are the key drivers of the evolution, T50C (4^th^ mutation) and T39A (7^th^ mutation), because each mutation has resulted in significant increase in catalytic activity.

Many previous studies have reported *in vitro* selection of ribozymes and DNAzymes from random-sequence DNA or RNA pools. To our knowledge, our study here presents the first example of turning a non-catalytic DNA sequence into a DNAzyme through *in vitro* evolution. In comparison to the random-library approach, our defined-sequence approach requires a longer evolutionary time, and the derived DNAzyme exhibits a weaker catalytic activity. Specifically, G2501, which only produces 10^3^-fold rate enhancement for RNA cleavage, was obtained after 25 rounds of selective enrichment. In contrast, Breaker and Joyce isolated an RNA-cleaving DNAzyme from a random-sequence pool after merely five rounds of selection, and the derived DNAzyme is capable of achieving 10^5^-fold rate enhancement for the same reaction^19^. Nevertheless, our study has demonstrated the feasibility to evolve a catalyst from a noncatalytic nucleic acid sequence.

At some point in prebiotic history, RNA must have taken on a catalytic role in order to self-replicate and catalyze other life-supporting chemistries, as described by the RNA World hypothesis. Our study shows that when an RNA-like polymer with a defined sequence is given a chance to mutate and reproduce, it can evolve into a purposeful enzyme. Our artificial evolution experiment was performed in a test tube in a few weeks whereas Mother Nature had millions of years to carry out her experiments. This begs the question of whether Mother Nature has explored a similar mechanism to patiently work her evolutionary magic to evolve powerful enzymes from noncatalytic polymeric molecules, like RNA, that were available in the prebiotic world.

## Methods

### Materials

All oligonucleotides were synthesized using standard phosphoramidite chemistry (IDT, Coralville, IA) and purified by 10% denaturing polyacrylamide gel electrophoresis (dPAGE). Their concentrations were determined spectroscopically. T4 polynucleotide kinase (PNK), T4 DNA ligase and Tth DNA polymerase were obtained from Thermo Scientific, BioBasic and Biotools, respectively, along with 10× reaction buffers. [γ-^32^P]ATP and [α-^32^P]dGTP were purchased from Perkin Elmer. All other chemicals were obtained from Sigma-Aldrich.

### *In vitro* evolution

For the first round of selection, 100 pmol of synthetic BTA1 (5′-TACGC AGTCA GTCAG TGTAC ATCTT TTCTA TCAAC CCCAA AACTT TGGCA CAATG AAGTG GGTGA CTTTT GGCTA ACTAC CCGAA CTTCA-3′) was 5′-phosphorylated by PNK (10 units) at 37 °C first with 10 μCi [γ-^32^P]ATP (20 min) and with 1 mM ATP (20 min). The phosphorylated DNA was ligated to 110 pmol of S1 (5′-ACTCT TCCTA GCGrGA GGTTC GATCA AGA-3′; rG: guanosine ribonucleotide) in the presence of 110 pmol of LT1 (5′-TGACT GCGTA TCTTG ATCGA-3′) and 5 U of T4 DNA ligase (50 μL; 23 °C; 60 min). The ligated S1-BTA1 was purified by 10% dPAGE and used for RNA cleavage reaction (25 μL; 23 °C; 4 h) in 1 × selection buffer (100 mM KCl, 300 mM NaCl, 15 mM MnCl_2_, 15 mM MgCl_2_, 55 mM HEPES, pH 7.5 at 23 °C). Following incubation at room temperature for 4 h, 1 μL of 0.5 M EDTA (pH 8.0) was added. Self-cleavage of S1-BTA1 (118 nt) would generate a 104-nt DNA fragment, which was purified using 10% dPAGE and subjected to DNA amplification by two consecutive polymerase chain reactions (PCR). PCR1 was performed in a 50-μL mixture containing 1 × PCR buffer, 0.2 mM each dNTP, and 1 U of Tth DNA polymerase, 2.5 μM each of forward primer FP1 (5′-TACGC AGTCA GTCAG TGTAC-3′) and reverse primer RP1 (5′-TGAAG TTCGG GTAGT TAGCC-3′). A small aliquot of the 1^st^ PCR product was further amplified using FP1 and RP2 (5′-AAAAA AAAAA AAAAA AAAAA-iSp18-TGAAG TTCGG GTAGT TAGCC-3′; iSp18 refers to 18-atom hexaethylene glycol spacer) as primers. RP2 contains a poly(A) tail separated by a non-amplifiable linker and thus the 2^nd^ PCR product contains a non-coding strand that is 20-nt longer than the coding strand. This facilitates the separation of coding strand by dPAGE. The second PCR mixture also contained 5 μCi α-GTP for the purpose of radiolabelling. The coding sequence from PCR2 was purified using 10% dPAGE and used as the enriched pool for the second round of selection. A total of 25 cycles of test-tube evolution were conducted.

### High-throughput sequencing

The PCR amplicons from Generation 1, 2, 4, 6, 8, 10, 12, 14, 16, 17–25 were sequenced using an Illumina Miseq DNA sequencer at the Farncombe Metagenomics Facility, McMaster University. Raw sequencing data was processed using Illumina’s Basespace online NGS platform to sort tagged sequence pools and output sequence data in FASTQ format. Paired-end reads were merged and trimmed of primers using PANDAseq 2.6^22^. Sequences containing less than perfect complementarity were discarded to minimize sequencing errors in the dataset. FASTA format trimmed sequences were dereplicated and tagged with copy number using USEARCH v7.0.1090_i86linux32^23^. Trimmed and dereplicated data was then imported into a MySQL 5.6.17 database and annotated with metadata such as mutation name, copy number and selection round. MySQL database queries were used to export sequence data and population level statistics for further analysis in Microsoft Excel 2010. Sequences comprising the limited sequence space used for the determination of mutation pathways were enumerated manually and then used to query the MySQL database for copy number data. Frequencies of each sequence for a given round were calculated in Excel from sequence copy number and total round populations. Sequences with fewer than three copies detected per round were not considered for pathway analysis to reduce spurious signals, weak signals and contamination of early sequencing pools with late round sequences.

### Activity analysis of G16, 19, 22 and 25 (Fig. 1c)

100 pmol of each radioactively labeled DNA pool-S1 construct was incubated in 1 × selection buffer for 1, 2, 4, 24, 48 and 72 h prior to dPAGE analysis.

### Determination of RNA cleavage rate constants of S1, G0 and 25 (Fig. 1d)

100 pmol of radioactively labeled S1, G0-S1 and G25-S1 were incubated separately in 1 × selection buffer for 14 days. An aliquot was taken out each day and combined with 2 × denaturing gel loading dye to quench the reaction. All the samples were analyzed by dPAGE and visualized using Typhoon 9200 (GE Biosciences). The fraction of cleavage was quantified using Image Quant software (Molecular Dynamics). The rate constants were determined by plotting the natural log of the fraction of uncleaved substrate at various reaction times. The negative slope of the resulting line, generated by a least-squares fit to the data is taken as the rate constant for RNA cleavage.

### Activity comparison of top 10 G25 sequences

100 pmol of each of the top 10 sequences in the G25 pool was chemically synthesized and ligated to S1. Each ligated construct was incubated in 1 × selection buffer for 24 h. All the samples were analyzed by dPAGE and visualized using Typhoon 9200.

### Activity comparison of truncated G2501 (Figure S3)

A series of mutated sequences of G2501 containing sequential 5-nt internal truncation were chemically synthesized and ligated to S1. Each ligated construct was incubated in 1 × selection buffer for 24 h. All the samples were analyzed by dPAGE and visualized using Typhoon 9200. The fraction of cleavage was quantified using Image Quant software and normalized by taking G2501 as 100.

### Activity of DNAzyme constructs in *trans* (Figure S4)

Cleavage was tested in *trans* (i.e. where the substrate and sequence are not connected) with various enzyme/substrate constructs. Each radioactive substrate was incubated with each enzyme (S:E = 1:50) for 24 h. All the samples were analyzed by dPAGE and visualized using Typhoon 9200.

**Figure 4:**
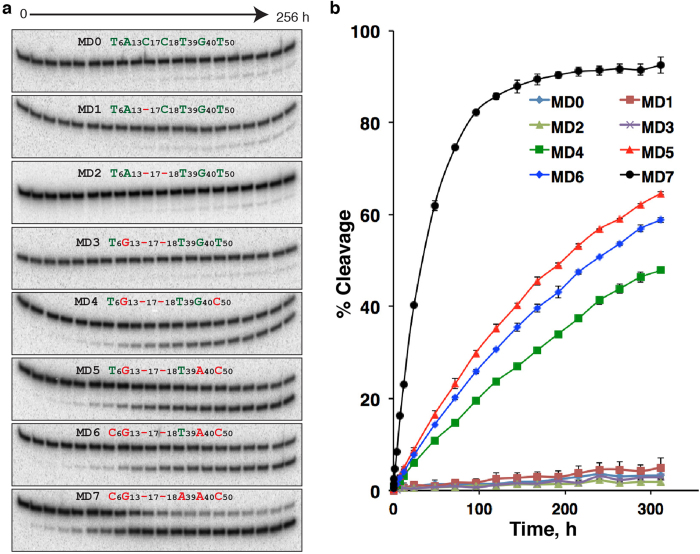
Activity analysis of proposed mutant DNAzymes along the evolution path. (**a**) The time-dependent cleavage activity of MD0-7. MD0 is G0, and MD7 is G2501. MD1-7 contain progressive mutations in the following sequential order: C17-, C18-, A13G, T50C, G40A, T6C and T39A. The time points (0–256 h) are set up to reveal both strong and weak activities expected of these DNA molecules. Cropped gels are used for better presentation and these gels were run under the same experimental conditions. The original uncropped gel images are provided in panel C of Figure S2 in the supplementary information. (**b**) Percent cleavage vs. reaction time.

Activity analysis of MD0-7 (Fig. 4). 100 pmol of each sequence was ligated to S1 and purified by dPAGE. Each purified construct was incubated in 1 × selection buffer for 0.5, 1, 2, 4, 8, 12, 24, 48, 72, 96, 120, 144, 168, 192, 216, 240, 264, 288, and 312 h. All the samples were analyzed by dPAGE and visualized using Typhoon 9200. The fraction of cleavage was quantified using Image Quant software.

## Additional Information

**How to cite this article**: Gysbers, R. *et al.* Evolution of an Enzyme from a Noncatalytic Nucleic Acid Sequence. *Sci. Rep.* **5**, 11405; doi: 10.1038/srep11405 (2015).

## Supplementary Material

Supplementary Information

## Figures and Tables

**Figure 1:**
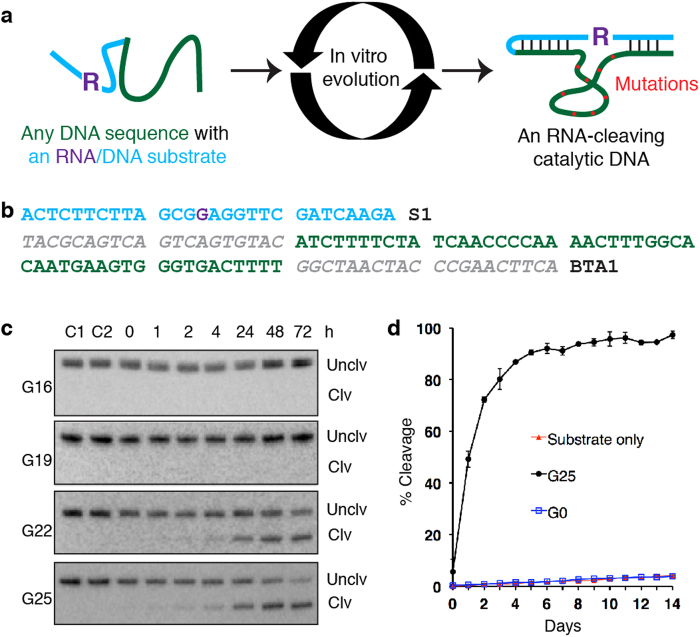
Evolving a catalytic DNA from a defined, non-catalytic DNA sequence. (**a**) The approach. An arbitrarily chosen, 90-nt single-stranded DNA sequence (green and grey) is joined to a chimeric DNA/RNA substrate (light blue) containing a single ribonucleotide as the cleavage site (R). This construct is then subjected to many rounds of selective amplification (using a selection scheme detailed in Figure S1), with a goal to turn the originally noncatalytic DNA sequence into an RNA-cleaving DNAzyme. It is expected that the evolved sequence will acquire crucial nucleotide mutations (red dots) that act as the catalytic switches. (**b**) Sequences of the substrate and the starting DNA sequence. The 28-nucleotide substrate sequence (S1) is designed at random. The single ribonucleotide, guanine ribonucleotide, is shown in purple. The candidate DNAzyme sequence, BTA1, is arbitrarily chosen to be the first 50-nt sequence (green) for the *Bos taurus* (cattle) albumin mRNA and two terminal 20 nucleotides (grey) located at both 5′ and 3′ ends as the primer binding sites for PCR. (**c**) Activity analysis of select pools established by *in vitro* selection. A total of 26 rounds of selective amplification (RNA cleavage for 4 hours; separation of cleaved product by denaturing polyacrylamide gel electrophoresis, dPAGE; PCR based amplification of purified cleavage product) were carried out. Upon the completion of *in vitro* evolution, four DNA generations, G16, G19, G22 and G25 were assessed for cleavage activity in time-dependent fashion. As a comparison, C1 and C2 show the activities of G2501 and the specified pool, respectively, incubated for 72 hours without added selection buffer. Cropped gels are used for better presentation and these gels were run under the same experimental conditions. The original uncropped gel images are provided in panel A of Figure S2 in the supplementary information. (**d**) The substrate, G0 and G25 were subjected to kinetic analysis for RNA-cleaving activity.

**Figure 2:**
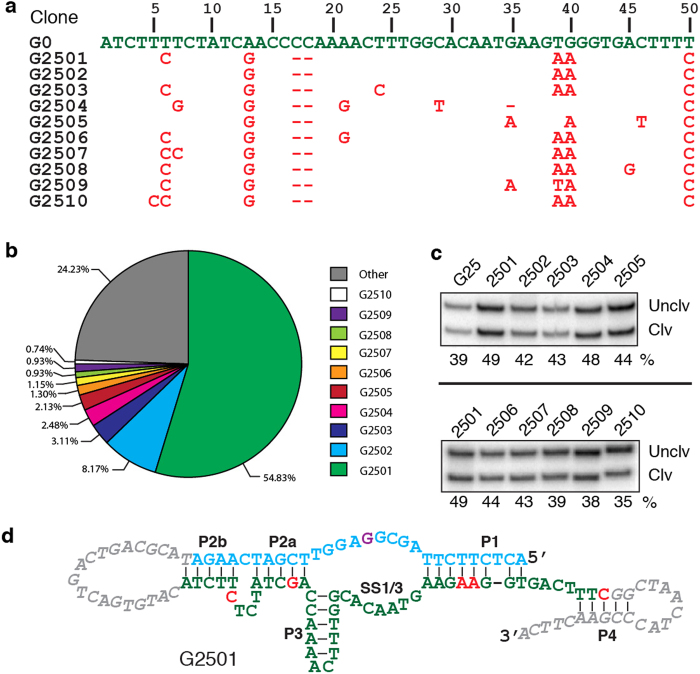
Catalytic DNA sequences from G25. (**a**) Sequencing results. The G25 pool was sequenced and the top 10 sequences are listed (without primer binding sites). The first entry is the original BTA1 (G0). Mutations and deletions (indicated by “-”) in each G25 sequence are shown in red; unmutated nucleotides are omitted for clarity. (**b**) A pie chart showing the percentage of each top 10 sequence in the G25 pool. (**c**) Comparison of top 10 sequences for cleavage activity. The reaction time is 24 h. The G25 pool was used as a control. Cropped gels are used for better presentation and these gels were run under the same experimental conditions. The original uncropped gel images are provided in panel B of Figure S2 in the supplementary information. (**d**) A putative secondary structure of G2501.

**Figure 3:**
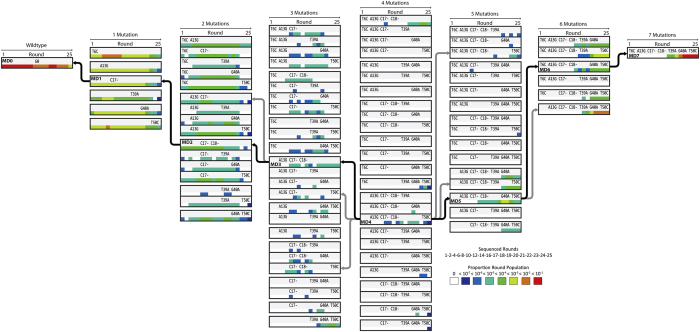
Identification of an evolutionary pathway. Mutants comprising the sequence space defined by the 7 mutations observed in G2501 are arranged by increasing mutation number across columns from left to right. Sequences are arranged by mutation position from 5′ (top) to 3′ (bottom) within columns. The proposed pathway is anchored at the 4^th^ mutation given the persistence of the A13G/C17-/C18-/T50C indicated by the darker shading. Arrows linking adjacent columns indicate mutants within a single mutation, black arrows indicate the favoured mutant. Color spectrum corresponds to the log binned proportion of each mutant within a round.
