# Supplementary material for: Evolution of an Enzyme from a Noncatalytic Nucleic Acid Sequence
Source: Sci Rep. 2015 Jun 19;5:11405. doi: 10.1038/srep11405 (PMC4473686; doi:10.1038/srep11405)
Supplement: Supplementary Information [file srep11405-s1.pdf]

Evolution of an Enzyme from a Noncatalytic Nucleic Acid Sequence

Supplementary Information

Rachel Gysbers[1,3†], Kha Tram[2†], Jimmy Gu and Yingfu Li[1,2,3]*

[1]Department of Biochemistry and Biomedical Sciences, [2]Department of Chemistry and Chemical Biology, [3]Origins Institute, McMaster University, 1280 Main St. W., Hamilton, ON L8S 4K1, Canada

[†]These authors contributed equally to this work.

*Email: liying@mcmaster.ca

A

S1: 5′-ACTCTTCCTA GCGGAGGTTC GATCAAGA

BTA1: 5′-*TACGCAGTCA GTCAGTGTAC* ATCTTTTCTA TCAACCCCAA AACTTTGGCA CAATGAAGTG GGTGACTTTT *GGCTAACTAC CCGAACTTCA*

FP1: 5′-TACGCAGTCA GTCAGTGTAC

RP1: 5′-TGAAGTTCGG GTAGTTAGCC

RP2: 5′-AAAAAAAAAAAAAAAAAAAA-L-GTAGTTAGCC

T1: 5′-TGACTGCGTA TCTTGATCGA

B

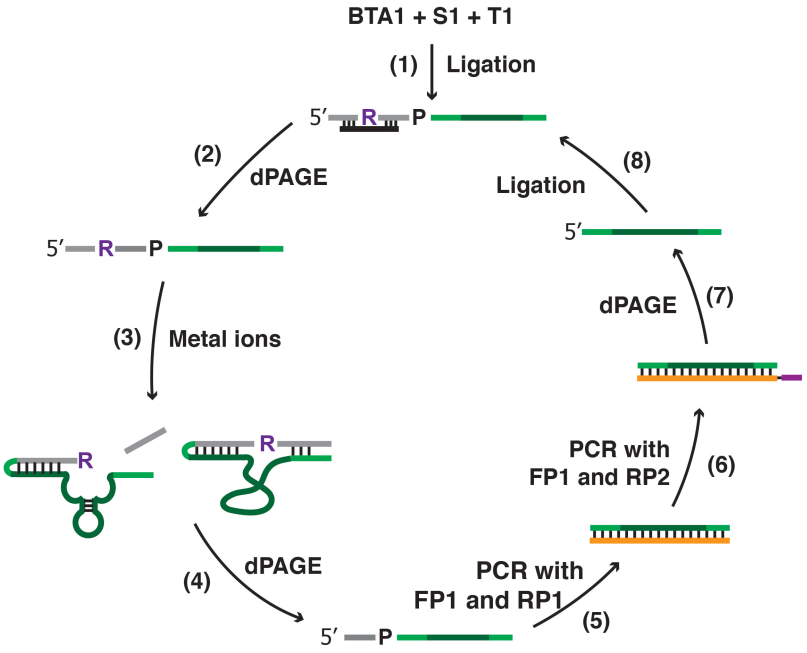

Figure S1. In vitro selection. (A) The sequences of the DNA molecules used for the study. (B) In vitro evolution scheme. (1) Ligation of BTA1 to S1 templated by T1; (2) purification of ligated S1-BTA1; (3) incubation of S1-BTA1 in the presence of metal ions; (4) purification of cleaved products by dPAGE; (5) PCR using FP1 and RP1 as primers; (6) PCR with FP1 and RP2 as primers (note: RP2 contains hexaethyleneglycol spacer (L, panel A) and A20 tail at the 5' end (the spacer prevents the poly-A tail from being amplified, making the non-DNAzyme-coding strand 20 nucleotides longer than the coding strand); (7) purification of BTA1 strand by dPAGE; (8) Phosphorylating BTA1 and ligating it to S1. The cycle of steps 2-10 was repeated for 25 times in this study.

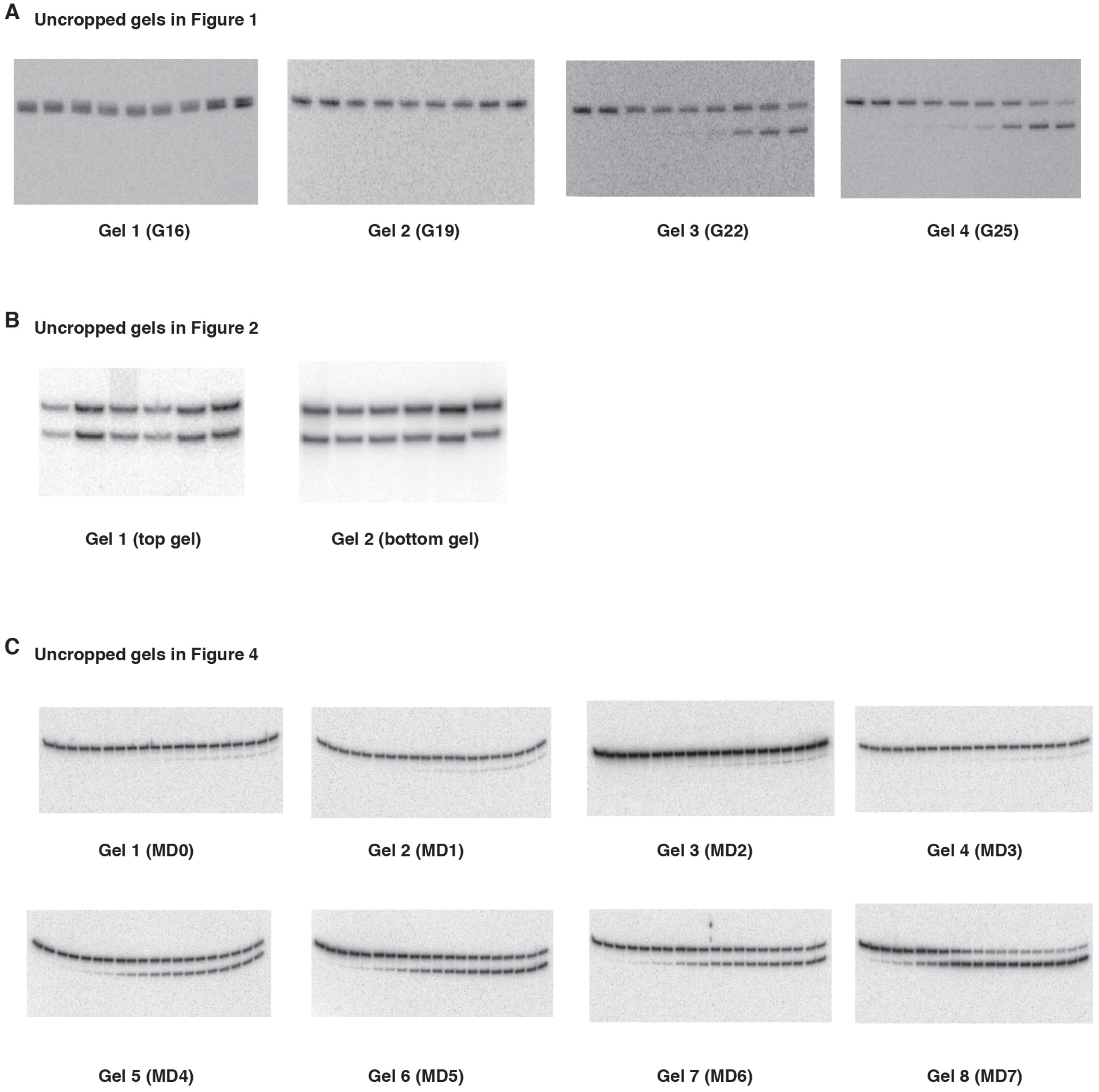

Figure S2. Original gel images for (A) Figure 1C, (B) Figure 2C and (C) Figure 4A of the main manuscript.

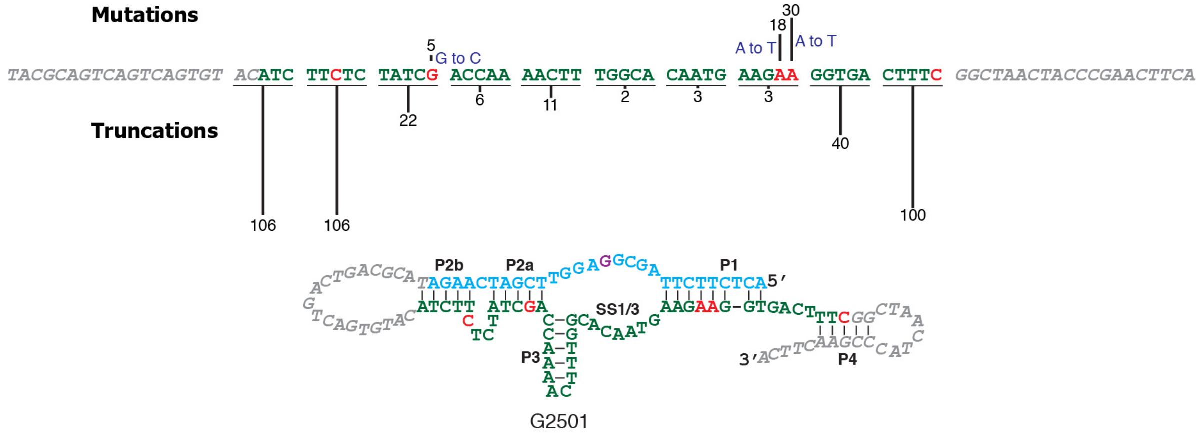

Figure S3. Verification of putative secondary structure of G2501 via nucleotide deletions and mutations. Activities of constructs with sequential 5-nucleotide deletion are shown beneath the sequence and those with a single nucleotide mutation are provided above the sequence. Relative activities in reference to G2501 (which is taken as 100) are shown.

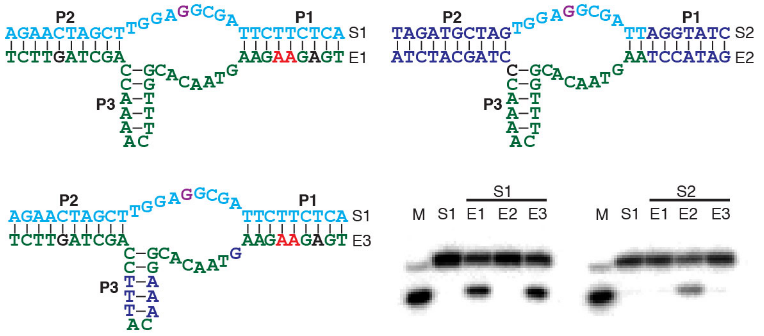

Figure S4. Simplified secondary structure of G2501 and verification of duplex elements within. Three structural variants were tested. The first structure represents a *trans*-acting construct with perfectly matched P1 and P2. The refined secondary structure of the DNAzyme is designed based on the structural model in Figure 2D and truncation data in Figure S2. Specifically, all catalytically disposable nucleotides are truncated; *c*is-acting construct has been made into *trans*-acting format; perfectly base-pairing P1 and P2 are created (with the incorporation of two black nucleotides). The second structure is the altered version of the first structure where significant nucleobase substitutions (dark blue base-pairs) are made within both P1 and P2 in order to change the sequence identity but maintain the duplex structure. The third structure is similar to the first one except that three base-pair co-variations (dark blue base-pairs) are made within P3 in order to change the nucleotide content of P3 but maintain its duplex structure. The PAGE gel shows the testing of the activity of matched and mismatched substrate (S1 and S2) and enzyme strands (E1, E2 and E3).
